# Post-Diphtheritic

**Published:** 1913-02

**Authors:** 


Post-Diphtheritic Paralysis of Pseudo-tabetic type, treated by antidiphtheritic serum, cure. B. Auche, ibid., October 13, 1912. The patient, aged 34, contracted diphtheria from his own child, who died. He received the usual treatment, including three injections of serum. The paralysis was noted at about the beginning of convalescence, between three and four weeks from the onset of the disease. A month later, he was admitted to the service of the authors and treated as noted, receiving in all, 140 c. c. of serum in a month. He was allowed to leave the hospital a trifle prematurely, but practically well, four months from the initial attack of diphtheria.

